# Time and timing in the acoustic recognition system of crickets

**DOI:** 10.3389/fphys.2014.00286

**Published:** 2014-08-12

**Authors:** R. Matthias Hennig, Klaus-Gerhard Heller, Jan Clemens

**Affiliations:** ^1^Behavioural Physiology, Department of Biology, Humboldt-Universität zu BerlinBerlin, Germany; ^2^Princeton Neuroscience Institute, Princeton UniversityPrinceton, NJ, USA

**Keywords:** acoustic communication, insects, crickets, auditory processing, computational neuroscience

## Abstract

The songs of many insects exhibit precise timing as the result of repetitive and stereotyped subunits on several time scales. As these signals encode the identity of a species, time and timing are important for the recognition system that analyzes these signals. Crickets are a prominent example as their songs are built from sound pulses that are broadcast in a long trill or as a chirped song. This pattern appears to be analyzed on two timescales, short and long. Recent evidence suggests that song recognition in crickets relies on two computations with respect to time; a short linear-nonlinear (LN) model that operates as a filter for pulse rate and a longer integration time window for monitoring song energy over time. Therefore, there is a twofold role for timing. A filter for pulse rate shows differentiating properties for which the specific timing of excitation and inhibition is important. For an integrator, however, the duration of the time window is more important than the precise timing of events. Here, we first review evidence for the role of LN-models and integration time windows for song recognition in crickets. We then parameterize the filter part by Gabor functions and explore the effects of duration, frequency, phase, and offset as these will correspond to differently timed patterns of excitation and inhibition. These filter properties were compared with known preference functions of crickets and katydids. In a comparative approach, the power for song discrimination by LN-models was tested with the songs of over 100 cricket species. It is demonstrated how the acoustic signals of crickets occupy a simple 2-dimensional space for song recognition that arises from timing, described by a Gabor function, and time, the integration window. Finally, we discuss the evolution of recognition systems in insects based on simple sensory computations.

## Acoustic signals carry information on different time scales

Communication signals of different modalities can exhibit static and dynamic components (Bradbury and Vehrencamp, 1998). Dynamic signals change over time, an attribute that is shared by signals directed at different sensory modalities, from visual and olfactory signals to acoustic signals. Even for human speech, the temporal component is an important information channel that is decoded with sub-millisecond precision over multiple time scales (Giraud and Poeppel, 2012; David and Shamma, 2013; Garcia-Lazaro et al., 2013). The acoustic signals of many species from arthropods to vertebrates and humans vary over time and carry information in their temporal dynamic. For all species with low resolution for carrier frequencies and thus poor spectral analysis it is the temporal domain in which information can be transmitted. Particularly sound (pulse) rates, sound onsets and durations are important features in signals from insects, fish, frogs or mammals (Rose and Capranica, 1984; Langner, 1992; Crawford, 1997; Gerhardt and Huber, 2002; Felix et al., 2011). Although we observe and describe many of these features in the acoustic signals, our understanding of the underlying feature detectors in a receiver is by far less advanced.

**Figure 1:**
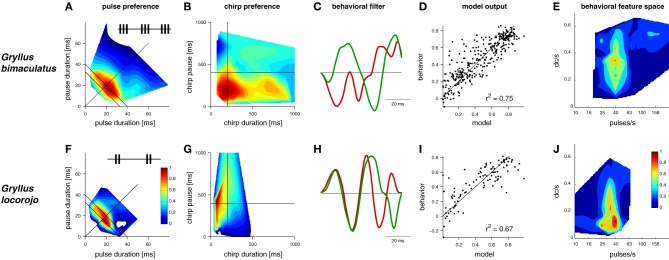
**LN-models derived from behavioral preference tests of two *Gryllus* species for acoustic signals**. Preference profiles for **(A,F)** pulse and **(B,G)** chirp patterns (insets: schematic song patterns). **(C,H)** linear filters (filters were scaled to unit-norm and have no units) **(D,I)** model output predicts behavior, units refer to the attractiveness of test patterns from behavioral trials with crickets and the respective output of the computational model **(E,J)** 2-dimensional feature space for song recognition, DC/s refers to the duty cycle per second (data in **A**,**B**,**F** and **G** modified from Grobe et al., 2012 and Rothbart and Hennig, 2012a, **C**,**D**,**E**, and **H**,**I**,**J** modified from Clemens and Hennig, 2013).

The songs of insects are a case in point. Over the last hundred years the calling songs of insects were recognized as important barriers for pregamic isolation and the song patterns were documented in numerous monographs (Otte and Alexander, 1983; Otte, 1994; Ragge and Reynolds, 1998). As insects usually have low spectral resolution it is the temporal component that carries most information. For most cases the long-range signals of insects reveal a comparatively simple signal structure (with the exception of grasshoppers and their courtship songs, cicadas with frequency modulations). Nevertheless there are now several examples that demonstrate that the information relevant for a receiver is distributed over several time scales, to which in insect songs we refer to pulses or syllables and chirps, trills or phrases (Figures 1A,F, Deily and Schul, 2009; Grobe et al., 2012). The physiological basis for a basic feature detector was demonstrated for grasshoppers (gap detection, Ronacher and Stumpner, 1988) and recently in crickets (pulse rate detector, Kostarakos and Hedwig, 2012). The central element of both is the timing of inhibition and excitation. However, the physiological correlate of a filter on a longer time scale is still elusive. It remains a challenge to understand the temporal computations on the longer time scale of chirps and phrases.

In crickets, but also katydids, it is known that females evaluate song signals on several time scales (Deily and Schul, 2009; Grobe et al., 2012). Especially, the songs of crickets are simple and binary-like as they are composed of single sound events, the pulses produced by the closing movements of the forewings (Huber et al., 1989). From an evolutionary point of view there exist several descriptions about the evolution of song patterns (Alexander, 1962; Otte, 1992, 1994; see also Desutter-Grandcollas and Robillard, 2003; Korsunovskaya, 2008). In these scenarios the ancestral calling songs consisted of long pulse trains, which were then modified by disruption into chirps and trills. Yet, it is unclear how the corresponding feature detectors of the receiver shaped the calling songs of crickets. We also lack an understanding of how feature detectors on short and long time scale changed during evolution and how these changes affected the calling song signals that can be observed today.

Recently, a simple solution was proposed for the evaluation of signals on different time scales (Figure 1, Clemens and Hennig, 2013). Two species of crickets within the same genus (*Gryllus bimaculatus*, *G. locorojo*) produce song patterns with short chirps. The females of both species evaluate the pulse rate on the short time scale (Figures 1A,F) and the chirp pattern on the long time scale (Figures 1B,G). A major difference between the two species lies in the preference for the chirp pattern (Grobe et al., 2012; Rothbart and Hennig, 2012a; see also Meckenhäuser et al., 2013). While one species accepted chirps over a wide range of duty cycles (Figure 1B), the other preferred chirps only over a small duty cycle range (Figure 1G).

**Figure 2:**
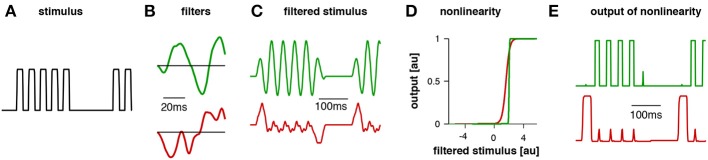
**Example traces for the model for *Gryllus bimaculatus***. The stimulus **(A)** had a pulse period of 40 ms (chirp duration 200 ms, chirp period 500 ms). The first filter (**B**, green) exhibited a pulse period of 40 ms and responded well to the pattern (**C**, green). The second filter's dominant modulation was relatively slow; accordingly, it responded poorly to this stimulus (**C**, red). For the computation of the filtered stimulus the filter **(B)** is first aligned with the beginning of the stimulus **(A)** and then multiplied with the amplitude values of the stimulus over the duration of the filter. The result is a product as a single point in time that reflects the similarity of the filter with the stimulus, high values indicating high similarity. The filter is then shifted by one step (given by the time resolution of the stimulus) and then multiplied with the respective amplitude values of the stimulus as before. This procedure is repeated until the end of the stimulus is reached and the filtered stimulus emerges as a new time series. Patterns were normalized such that the distribution of amplitudes over the whole stimulus set exhibited zero mean and unit standard deviation. The nonlinearity for each filter was relatively steep **(D)**, transforming the stimulus into an almost binary trace **(E)**. This trace was then integrated over time and the resulting values for each LN model were weighted to obtain a prediction of the phonotaxis score (modified from Clemens and Hennig, 2013).

These phonotactic responses can be reproduced using a general model that has 4 components: (1) a linear filter, (2) a nonlinearity, (3) an integration time window, and (4) a weighting function (summarized in Table 1). Linear-nonlinear (LN)-models are commonly used to describe the computations performed by sensory neurons (e.g., Pillow et al., 2008). These LN models exhibit a linear part, the filter, and a nonlinearity characterized by a threshold and a saturation (Clemens and Hennig, 2013). The linear filter describes the temporal tuning of the model—positive and negative lobes of the filter can be produced by precisely timed excitatory or inhibitory inputs to a neuron. The filter constitutes a template, which is compared to the stimulus pattern; the filter's output as given by the filtered stimulus thus corresponds to the similarity between the stimulus and filter (Figures 2A–C). For the evaluation of phonotactic responses, the output of LN-models for a test pattern was integrated over a time window of 1 s into a single feature value (Clemens and Hennig, 2013). This corresponds to the computation performed by integrator neurons used in drift-diffusion models of decision making and found in vertebrate cortex (Brunton et al., 2013). Song signals were processed in parallel by several LN-models, whose integrated output was linearly weighted to yield the predicted phonotaxis value for the test pattern (Figure 2).

**Table 1:** **Computational steps for pattern recognition using LN-models: the computational goals, the algorithms of computation and possible physiological implementations**.

	**Linear filter *L***	**Nonlinearity *N***	**Integration *I***	**Weighting function *W***
Computational goal	Preference function for temporal selectivity	Adjustment of temporal selectivity	Sampling/integrate over time	Tuning/sharpening of preference function
Algorithm	Gabor function	Sigmoidal function	Integration time window	Linear weight
Physiological implementation	Relative timing and strength of excitation and inhibition, intrinsic properties	Threshold and saturation	Synaptic facilitation/depression	Synaptic weights of excitation/inhibition
	Temporal coding>>>		>>>Rate coding	

Fitting this model structure to behavioral data showed that two LN-models sufficed to predict female responses, if correctly weighted against one another (Figures 1D,I, see Clemens and Hennig, 2013, for details). Accordingly, two linear filters emerged for each species (Figures 1C,H). Notably, recent recordings from individual brain neurons in crickets (Zorović and Hedwig, 2011; Kostarakos and Hedwig, 2012) appear be similar to the output predicted by LN-models (Figure 2E). At least one of these filters resembled a Gabor function; that is, a sinusoid with a given frequency modulated by a Gaussian distribution (Figures 1C,H, 2, see filter in green). Gabor filters are known from many systems and have been used to describe the feature selectivity of sensory neurons in the visual and auditory system. Interestingly, Gabor functions have emerged as optimal filters for efficient representations of natural stimuli (Olshausen and Field, 1996; Smith and Lewicki, 2006). These studies mainly studied generic stimulus representations with the objective of transmitting maximal information with the minimal amount of neural activity in a sparse code. In the context of song recognition, they serve to reproduce the high behavioral specificity for relatively simple, repetitive patterns.

Computing the preference functions of crickets with LN-models also offered a simple solution to the question of different time scales. Since the filters used were short (64 ms) temporal features on the long time scale were not explicitly filtered. Rather, selectivity for the chirp pattern resulted from integrating the song signal over time. The outcome of this integration depends on the overall energy of the signal and thereby sets the preferred duty cycle, the preference for which is indeed different between the two species (Figures 1B,G). Correspondingly, a new preference space for features on the song signals of crickets can be constructed (Figures 1E,J), that relies on temporal information on one axis (pulse rate in Figures 1E,J) and energy on the other (duty cycle per second in Figures 1E,J).

The computation with LN-models of preferences measured from female crickets produced a surprisingly simple view of the acoustic recognition system. In essence, recognition relies on the differentiating and integrating properties in the auditory pathway of crickets. Linear filters exhibit differentiating properties since they possess positive and negative lobes and can thereby be tuned to song features on the short time scale like the pulse rate; the integration reduces the output of the LN-models to a single value and can convey selectivity for song features on the time scale of the chirp—mainly the overall duty cycle or energy of the signal. Notably, the differentiating filter part of an LN-model can be understood by the relative timing of excitation and inhibition as it was recently observed for single brain neurons in crickets (Kostarakos and Hedwig, 2012). The simple integration step over time also offers a solution to a well-known paradox observed many years ago, when Pollack and Hoy reported the preference of female cricket for randomized and thus irregular calling songs (Pollack and Hoy, 1979). Indeed, it is an often observed feature of calling songs of crickets in North-America that males drop single pulses and therefore produce irregularities in their songs (Alexander, 1957, 1962; Desutter-Grandcollas and Robillard, 2003). However, if crickets just integrate over time, the specific timing is less important for song recognition.

The combination of precise timing and integration in our framework is a general feature of all decision-making processes. Therefore, the conclusions drawn in the context of insect song recognition potentially are of much broader relevance. Standard models of perceptual decision-making consist of a feature detection stage, which extracts so-called “sensory evidence,” and an integration stage, which accumulates the sensory evidence in a decision variable. In the feature detection stage (our LN-model), behavioral selectivity for short and precise temporal patterns can be implemented. In contrast, the integration step leads to a potential invariance to the exact time of occurrence of these patterns while conveying selectivity for large-scale features of the stimulus (cf. McDermott et al., 2013).

From these observations, three question on processing and recognition of temporal patterns arise that we aim at in the following sections:

1. What is the role of time and timing for analysis i.e., which types of preference functions can be created by LN-models with Gabor-functions as feature detectors and is there evidence that these perceptual spaces exist?
2. How do feature detectors operate? How do combinations of excitation and inhibition—if modeled as Gabor functions—affect preference functions? How can Gabor functions transform phenotypic preference profiles during a speciation event? What is their physiology?
3. Is there a more general scheme of sensory processing to which LN-models with their properties of time and timing conform?

## LN-models as feature detectors: properties of time and timing

The general model used for recognition of insect song signals has 4 components, a linear filter (L), a nonlinearity (N), an integration (I) and a weighting function (W, Table 1). The linear filters found for insect songs bear a striking similarity with Gabor functions, a property that makes them very attractive for a more general approach to auditory processing in insects (Clemens and Hennig, 2013; Clemens and Ronacher, 2013). Gabor filters offer a useful and simple tool for parameterization of the filter part that can also be implemented easily by general neuronal mechanisms such as the pattern of excitation and inhibition. Therefore, Gabor-filters served successfully in the past to model sensory processing in different modalities (e.g., visual: Priebe and Ferster, 2012, auditory: Smith and Lewicki, 2006). The complete model is abstract, since we use behavioral data for our calculations. Therefore, we can make no specific predictions about whether any given component is implemented in the physiology of a single cell. In principle, the properties of the model can be distributed over many cells and many parallel computations. Likewise we can make no predictions about biophysical implementations by specific ion channels or synaptic receptors. If, however, the filter component was implemented in a single cell, it is possible to predict the specific input patterns of excitation and inhibition to such a cell or its intrinsic properties from the positive and negative lobes of the filter function (see Table 1). Here, we examined how the linear filter described by the different parameters of a Gabor-function affects preference functions and tested whether it is possible to predict preference functions for acoustic signals in crickets and katydids by variation of linear filters.

**Figure 3:**
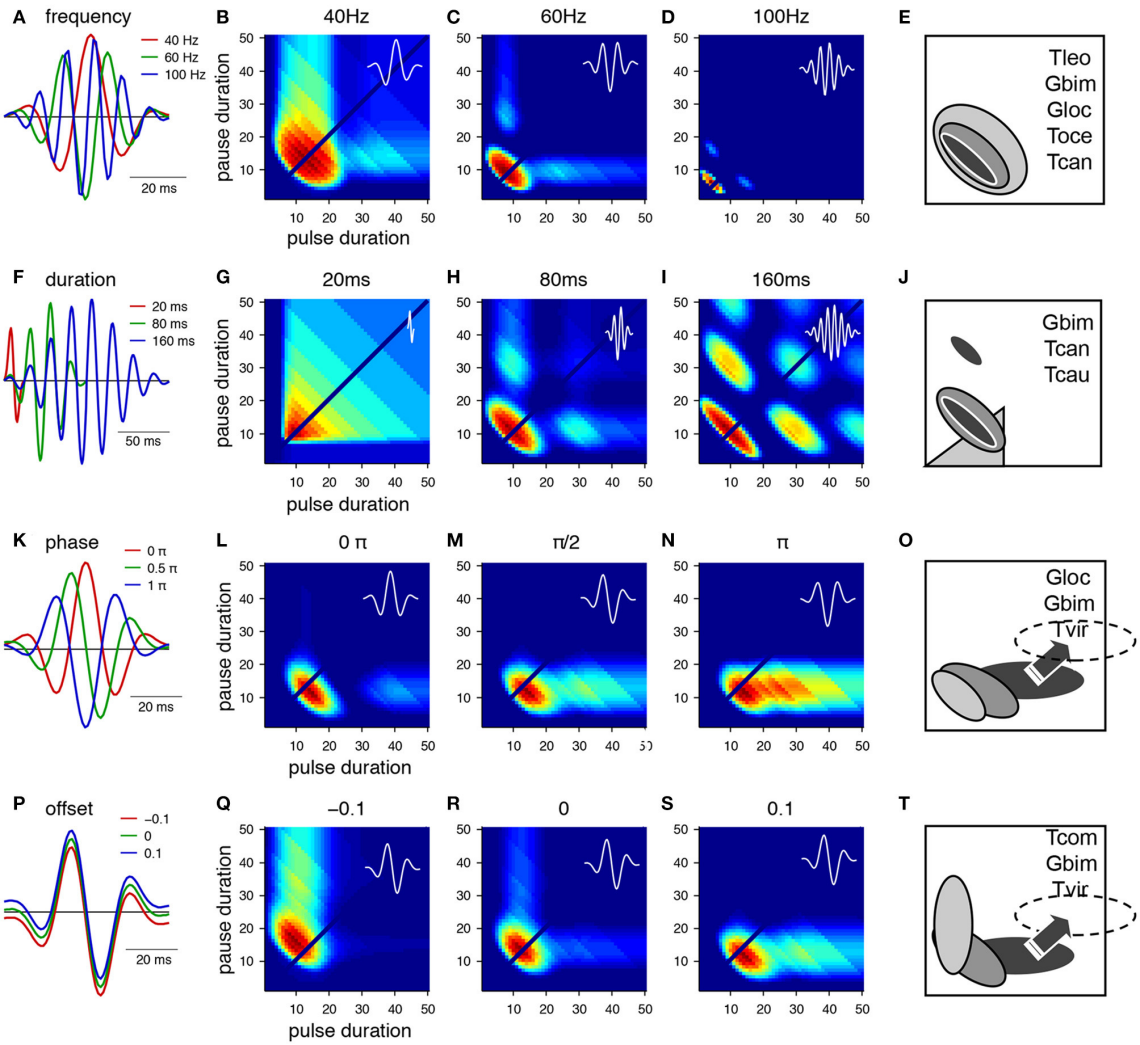
**From Gabor filters to behavioral preference functions for a pulse pattern**. **(A–E)** Variation of the frequency and resulting preference profiles. **(F–J)** Variation of filter duration. **(K–O)** Variation of phase. **(P–T)** Variation of offset. Shaded profiles on the right indicate known preference functions for pulse patterns in crickets and katydids. The pulse profile of *Tettigonia viridissima*, Tvir, was shifted from its original position (see arrow, dashed area). Species: Gbim: *Gryllus bimaculatus*, Gloc *G. locorojo*, Tleo *Teleogryllus leo*, Toce *Teleogryllus oceanicus*, Tcan *Tettigonia cantans*, Tcau *Tettigonia caudata*, Tvir *Tettigonia viridissima*, Tcom *Teleogryllus commodus*, see text for references. For calculation of the preference functions, pulse trains with different combinations of pulse durations and pause durations were created (chirp period 500 ms, chirp pause 250 ms). Each stimulus was filtered with the function shown in the first row **(A,F,K,P)** and passed through a sigmoidal nonlinearity (c.f. Figures 2D,E). The phonotaxis value was taken as the integral output of the nonlinearity (for details see Clemens and Hennig, 2013).

The main parameters that specify a Gabor function are the frequency, the duration, the phase and an offset (Figure 3). The intrinsic frequency mainly affects the preference for pulse rate. However, there is an additional effect as the frequency also changes the width of the preference function. The profiles are wider at low frequencies (Figure 3B) and more narrowly tuned at high frequencies (Figure 2D). Corresponding examples of wide preference profiles for low pulse rates (Figure 1E, *T. leo*, Rothbart and Hennig, 2012b), intermediate profiles (*G. bimaculatus* Hennig, 2009, *T. oceanicus* Hennig, 2003; Hennig, *Tett. cantans* Schul, 1998) and narrow profiles (*G. locorojo*, Rothbart and Hennig, 2012a) exist. Physiologically, the frequency can be set by the relative timing of excitation and inhibition or by oscillatory properties of subthreshold conductances (Hutcheon and Yarom, 2000; Schreiber et al., 2004).

**Figure 4:**
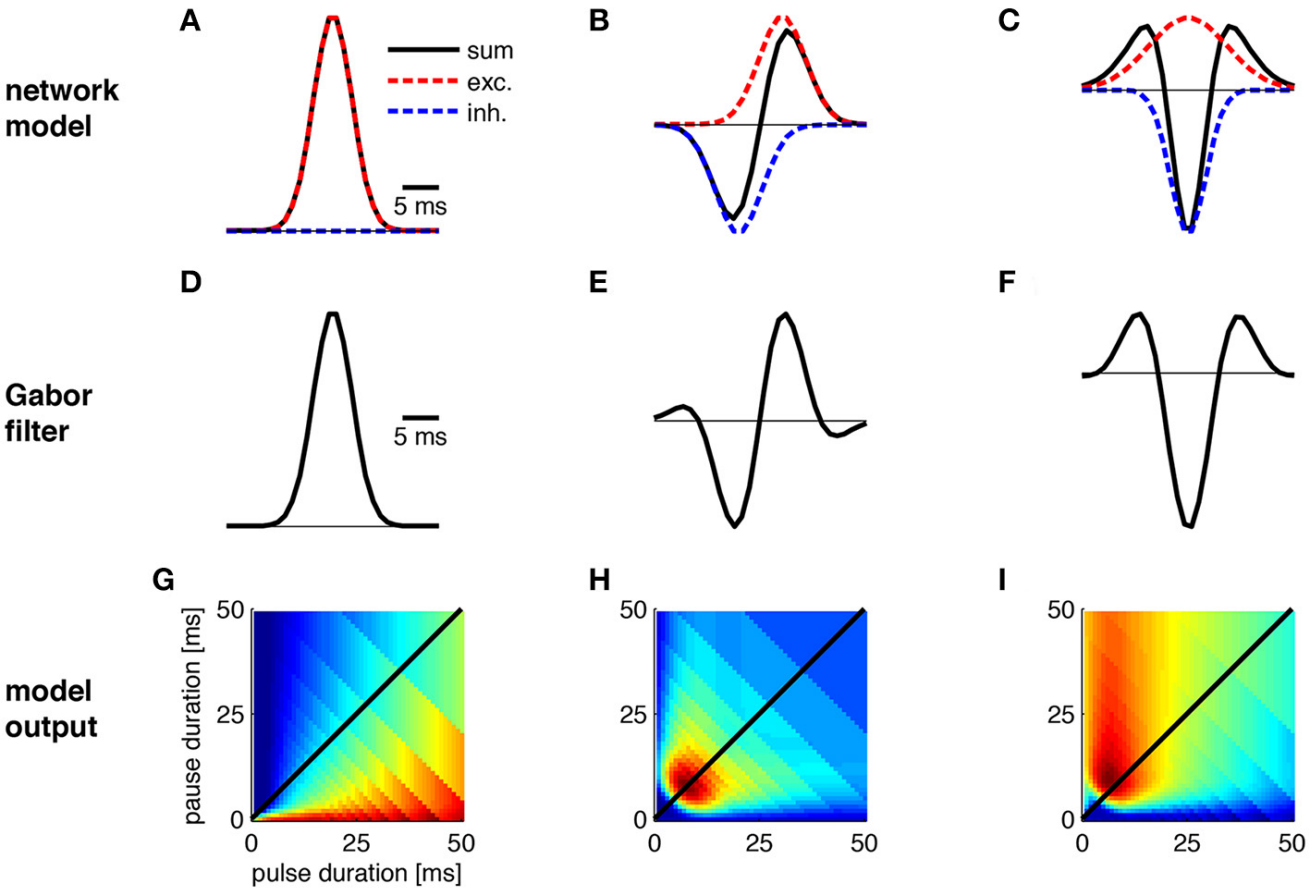
**Transitions between Gabor functions and corresponding changes in pulse profiles**. The physiological correlate of these transitions was explored using a simple network model in which a cell integrates inhibitory and excitatory input. **(A–C)** Gabor functions emerge as a result of excitation (red) and inhibition (blue) in a network model. **(D,E)** Shape of Gabor filters from **(A–C)**. **(G–I)** Preference for pulse patterns as derived from the Gabor filters in (**D–F)**. Note that the nonlinearity used in **(I)** differs from the one in Figure 3N. See Figure 3 for calculation of preference profiles.

The duration of the Gabor function at a given frequency mostly affects the width of tuning for pulse rate (Figures 3G–I). At longer durations the filter accommodates several oscillations and therefore responds at lower multiples of the preferred pulse rate (Figures 3H,I). This leads to the emergence of preference peaks at multiples of the preferred period (Figures 3H,I, i.e., 20, 40, 60 ms at low and high duty cycles). Convincing evidence for resonant properties stems from tettigoniids (Figure 3J, *Tett. cantans*, Bush and Schul, 2006; Webb et al., 2007, *Neoconocephalus affinis*, Bush et al., 2009, and *N. triops* Schul et al., 2014), although only the peak at low duty cycles was observed. At very short durations relative to the frequency the Gabor filter becomes single-lobed. It then exhibits a wide preference for duty cycle (Figure 3G) as the filter will respond to a whole range of pulse patterns and therefore becomes less precise for temporal properties of the pattern. The closest known match to a duty cycle preference stems from tettigoniids (see also Figures 4D,G, *Tett. caudata*, Schul, 1998).

The phase of the Gabor function at a given duration and frequency will mostly affect the range of different pulse durations to which the filter responds (Figures 3L–N). The change of phase extends the preference range along the pulse duration axis and does not affect the selectivity for pause duration (Figures 3L–N). Examples for such preference profiles stem partly from the cricket *G. bimaculatus* (Figure 1A) and the tettigoniid *Tett. viridissima* that exhibits a clear preference for pause durations over a wide range of pulse durations (Figures 3N,O, Schul, 1998, note that the profile of *Tett. viridissima* was shifted to appropriate pause durations in the preference panel). The different phases can also be viewed as a change in the relative timing of excitation and inhibition that is the lobes above and below zero (Figure 3K).

A change in offset of a Gabor function results in a rotation of the preference profile that remains centered at a particular period i.e., pulse rate (Figures 3Q–S). An extension of preferences along the pulse or the pause duration axis is observed and corresponding examples of preference profiles are known (Figure 3T, *T. commodus*, Hennig, 2003, *G. bimaculatus* Hennig, 2009, *Tett. viridissima* Schul, 1998). Physiologically, an offset corresponds to a tonic excitation or inhibition as the mean of the Gabor function is above or below zero (Figure 3P). A shift of the nonlinearity toward lower or higher thresholds can have similar effects on the preference profiles as the positive or negative offsets of the filter.

The preference profiles from Figure 3 serve to illustrate how quantitative changes of the parameters that specify a Gabor function will modify the range of accepted pulse patterns. A comparison of these profiles (Figure 3) shows that different parameters may yield the same or similar preference ranges (e.g., in Figures 3B,Q; Figures 3M,N,S). From an evolutionary perspective there are different dials at a Gabor filter that may be turned to produce the same result. Besides the preference range specified by the parameters of the Gabor function, the sigmoidal nonlinearity provides an additional degree of freedom to change the output of an LN-model (Clemens and Hennig, 2013). In the case of simple pulses as in cricket songs, the important component of the nonlinearity lies in the threshold function that affects the value of the integral (Table 1). The nonlinearity then serves to adjust the temporal selectivity without changing the principal shape of the profile determined by the Gabor function (Table 1, Clemens and Hennig, 2013).

The different preference profiles arose by modifications of the abstract parameters of the Gabor functions (Figures 3A,F,K,P; Table 1). Since the phenotypic preference profiles of known sibling species differ markedly (e.g., *Teleogryllus oceanicus* and *T. commodus* in Figures 3E,T, *Tettigonia cantans* and *Tett. caudata* in Figure 3J), the underlying neural circuitry is expected to change within the short evolutionary time spans required for speciation. To explore the changes in circuit parameters that could give rise to these different preference profiles, we used the most simple network model that could produce Gabor-like filters in its output by combining excitatory and inhibitory inputs (Figure 4). The simplest, uni-lobed Gabor filter can by constructed using only excitatory inputs to a particular neuron without an inhibition (Figures 4A,D). Such a Gabor filter will respond well to pulse trains composed of different pulse durations and pauses that exhibit a high duty cycle and therefore this filter resembles a duty cycle detector as observed for *Tett. caudata* and *N. robustus* (Figure 4G; Deily and Schul, 2004). The operation of a duty cycle detector corresponds to an integration of the input signal. By addition of a preceding inhibitory input of the same strength as the excitation a typical, multi-lobed Gabor filter emerges as output of a neuronal network (Figures 4B,E,H) that exhibits the frequently observed pulse rate preference (Figure 3). Similar patterns of synaptic input are known from recordings of single neurons in the brain of crickets that also exhibit the corresponding preference profiles (Kostarakos and Hedwig, 2012). The operation of such a pulse rate detector corresponds to a differentiation of the input signal, since the corresponding Gabor filter will only respond to pulse pairs with the correct pulse period, i.e., the inverse of the pulse rate. Delaying the inhibitory component as well as increasing its amplitude relative to the excitation (Figure 4C) will then produce a Mexican-hat-like Gabor filter with a strong negative lobe (Figure 4F) that will give rise to a preference for a particular range of pulse durations (Figures 3P, 4I). Thus, simple and testable changes in the timing and strength of excitation and inhibition can transform a behavioral preference function from a simple energy detector to a pulse rate detector and a pulse duration detector (Figure 4). In addition to the minimal network model (Figure 4), there are other ways for the physiological implementation of Gabor filters. As well as the timing of excitation and inhibition, post-inhibitory rebound excitation has been found to contribute to period selectivity (Large and Crawford, 2002). Changing the expression levels of the conductances underlying rebound spiking (Felix et al., 2011) could be an alternative strategy for tuning a behavioral preference function during speciation events. Independent of their specific implementation that can be tested experimentally, Gabor filters offer a parsimonious explanation for evolutionary transitions between phenotypically different preference functions by changes in synaptic parameters or in intrinsic properties.

## General properties of LN-models

The description of phenotypic preference profiles by LN-models (Figures 3, 4) allows placing the computations within the auditory pathways of crickets in a more general framework of sensory processing (Table 1). Barlow (1961), Marr (1982) and Konishi (1990) emphasized the importance of understanding the computational goals and implemented algorithms for our understanding of sensory processing in different modalities and sensory pathways. The general computational goal of auditory processing of crickets and katydids for mate recognition is specified by the recognition of the conspecific song signal or, more parsimoniously by the discrimination of its own signal against all other signals (c.f. Gerhardt and Huber, 2002). For each component of a LN-model it is also possible to specify a computational goal and an appropriate algorithm (Table 1). From computational goals and algorithms the salient cues (Konishi, 1990) for signal discrimination can be derived that consist of temporal information given by pulse rate and duty cycle (Figures 3, 4).

The extraction of these salient cues is achieved by specialized circuits that represent a physiological implementation of the linear Gabor filters, given by the timing and strength of excitation and inhibition (Figures 3, 4), combined with their respective nonlinearity (Table 1, Clemens and Hennig, 2013). The other components required for signal discrimination within this framework, the integration time window and the linear weighting function, may be represented by general properties known from synaptic transmission and dendritic integration (Table 1). Therefore, the physiological implementations of LN-models can be derived from intrinsic properties of neurons, their synaptic input patterns or small neural networks (Figure 4, Table 1). Although examples exist that suggest single neurons can exhibit the properties of individual LN-models (see examples in Clemens and Hennig, 2013), the models derived from behavioral preference functions reflect the abstract output of the whole system and it is not explicitly necessary to observe the convergence at the single cell level (see Clemens and Ronacher, 2013). The sequence of processing from LN-model to integration and weighting also involves a transformation of coding with high temporal precision to a rate code (Table 1). Such transformations are known from the auditory pathways of insects (Schildberger, 1984; Vogel et al., 2005; Clemens et al., 2011; Kostarakos and Hedwig, 2012) and correspond to the more general scheme also known from vertebrates (Joris et al., 2004).

In summary, Gabor-functions as the basic, linear part of LN-models provide a unitary and simple way for understanding diverse preference functions of crickets and katydids. Conceivable evolutionary changes and transitions between preference profiles of sibling species can be derived easily from small changes of properties (i.e., frequency, duration, phase, offset of Gabor functions, Figures 3, 4). Preference functions with qualitatively different phenotype can be transformed drastically by change of a single parameter (Figures 3, 4). In order to better understand such transitions it is a principal requirement to measure preference functions of insects for acoustic signals not only as a pulse profile but also with respect to the energy preference for a given time window of integration (Figure 1).

## The discriminative power of LN-models

**Figure 5:**
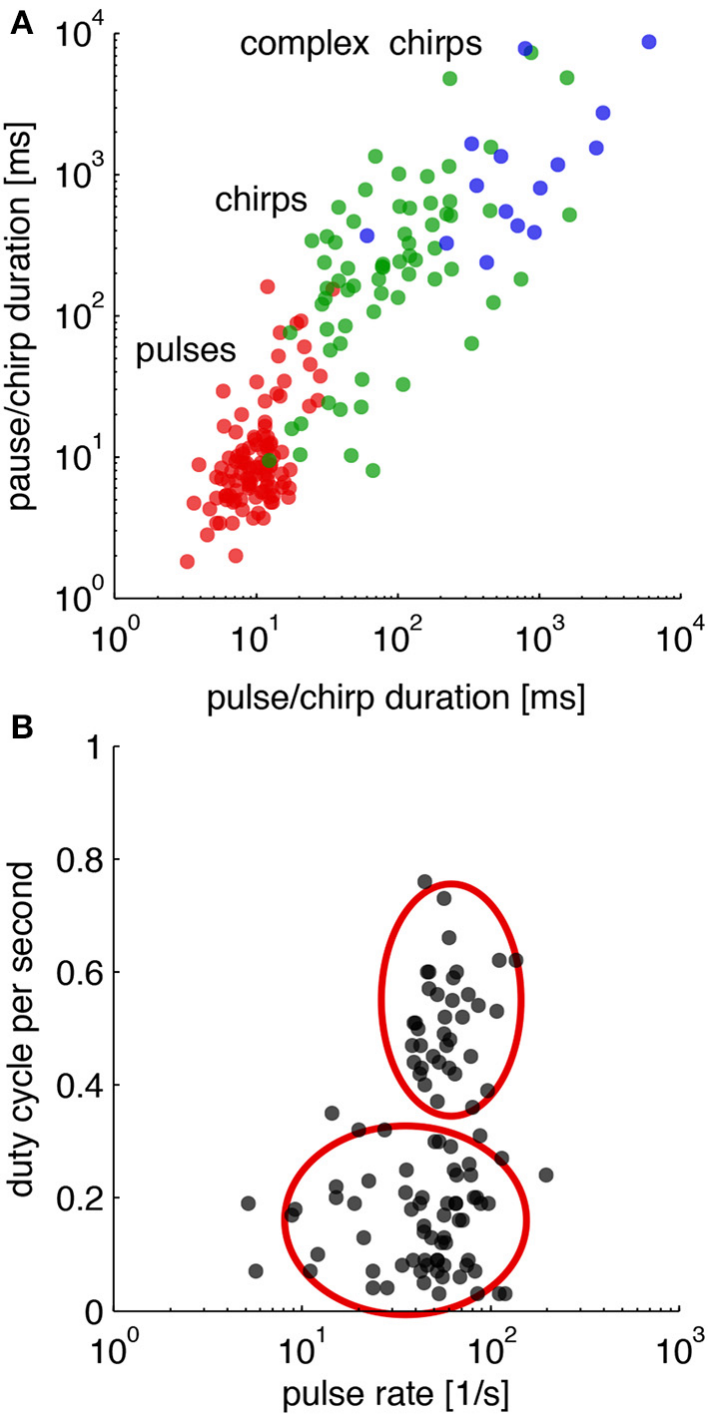
**Temporal parameters in the songs of crickets**. **(A)** Distribution of temporal parameters in the calling song for crickets on different time scales. **(B)** Distribution of calling songs in a 2-dimensional feature space (duty cycle per second refers to the normalized integral i.e., the energy of the song envelope). Cricket songs exhibit mostly lower or higher energy equivalent to songs with short chirps and long trills (red ellipsoids). Calling songs of 106 species of crickets from http://entnemdept.ufl.edu/walker/buzz/cricklist.htm. Song recordings were rectified and low-pass filtered (200 Hz) to compute a smooth envelope. Temporal measures such as pulse and chirp durations and pauses were obtained by a threshold function (see Grobe et al., 2012, for details).

There are only few components of which LN-models are built (Table 1). Although the songs of crickets differ in pulse rates, chirp rates and chirp durations as specified by the number of pulses in a chirp, there are many known similarities within and between different genera (Alexander, 1962; Otte, 1992). How sufficient are these differences between species for song discrimination by LN-models? To evaluate the discriminative power of LN-models, we first surveyed the songs of more than 100 species of crickets in 7 genera (http://entnemdept.ufl.edu/walker/buzz/cricklist.htm). The envelopes of the song patterns were analyzed for their temporal parameters on several time scales (Figure 5). The shortest unit of time is given by the pulse rate (red dots in Figure 5A). Longer temporal units are observed in the chirp pattern that may consist of simple chirps or complex pulse trains as for instance seen in chirps build from a series of shorter double or triple pulse trains (green and blue dots in Figure 5A). From a more traditional point of view, some species of crickets would be expected to analyze the song pattern at least on two time scales, that of the pulse and the chirp pattern (Figure 5A). Especially the complex song patterns may require sophisticated processing on several time scales. A transformation of the song patterns into a 2-dimensional feature space as suggested by LN-models (Figures 1E,J) shows that only two computations are required; an extraction of the pulse rate and a measurement of the energy component (Figure 5B). The songs of crickets in North America then show a common pulse rate between 30 and 100 pps with only few exceptions (Figure 5B). The distribution of energy in the songs of those crickets was largely bimodal, suggesting that songs of crickets can be roughly divided into two groups with shorter and longer chirps (Figure 5B).

**Figure 6:**
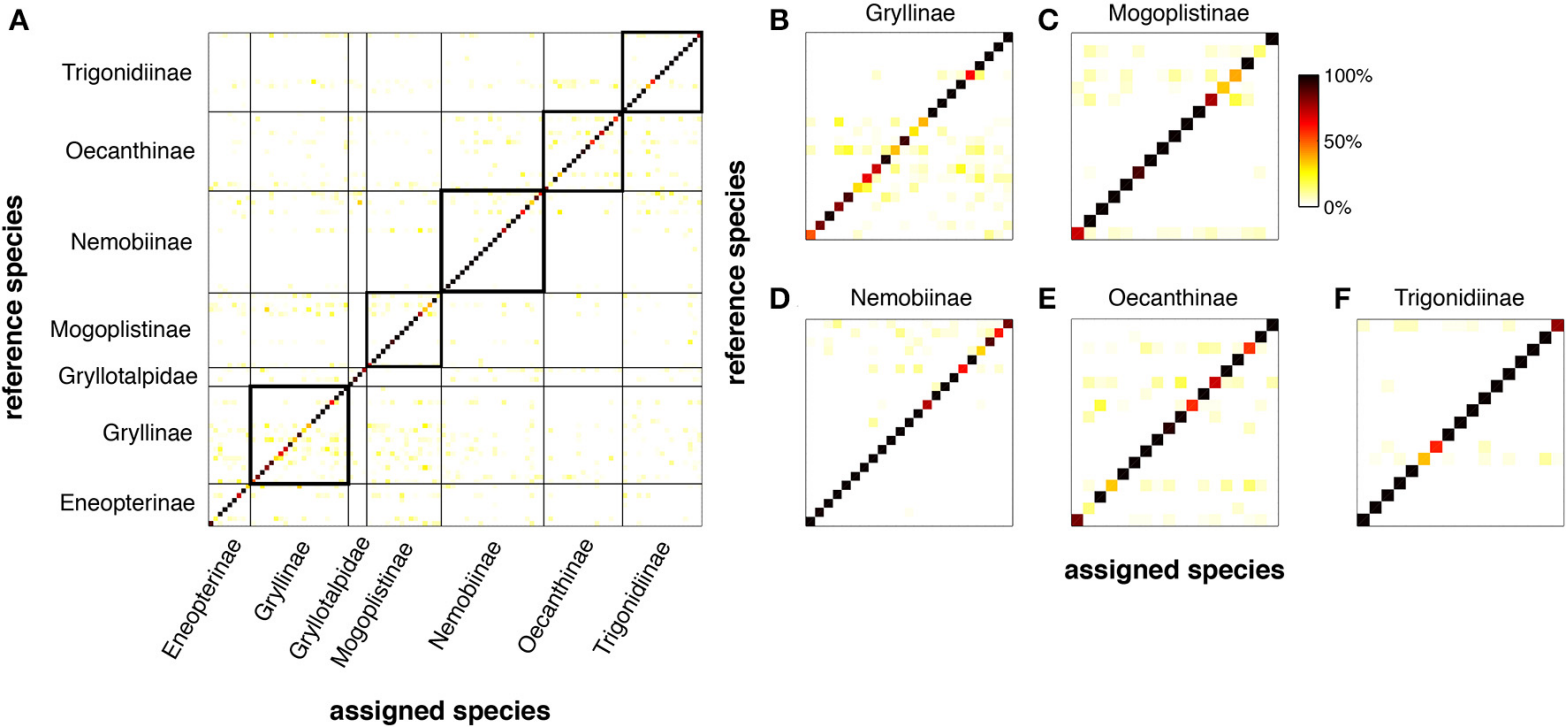
**LN-models discriminate the songs of 106 North-American species of crickets**. For each species, we trained a model with two filter-nonlinearity pairs (see Figures 1, 2) to discriminate its own song from all the other 105 songs using a Genetic Algorithm (cf. Clemens and Hennig, 2013). Each row in **(A–F)** depicts the normalized output values of each species' model for all songs (color coded). Dark shading confined to the main diagonal indicates high specificity (i.e., phonotaxis scores) of the model output for the conspecific song (see color bar **C**). **(A)** Discrimination matrix (shading indicates predicted output value of the reference species for the tested species quality of correct assignment). **(B–F)** Discrimination matrix for 5 major subfamilies. Discrimination power was determined from song envelopes (low-pass filter: 200 Hz) by LN-models. Calling songs of crickets and taxonomical classification from http://entnemdept.ufl.edu/walker/buzz/cricklist.htm.

However, the question arises whether the power of LN-models is sufficient to discriminate between the songs of different species given that many of the song patterns show similar pulse rates (Figures 5A,B) or energy distributions (Figure 5B). We trained an LN model for each species to discriminate the conspecific song from all other song patterns (*N* = 106, see Clemens and Hennig, 2013, for Methods). This analysis reflects an unrealistically hard scenario, since the geographical distributions do not require that crickets discriminate their own song from more than 100 other cricket songs (http://entnemdept.ufl.edu/walker/buzz/cricklist.htm). Still, the discrimination of the song pattern for a particular species was remarkably high (Figure 6, median of correctly assigned species was 93%, chance level 1/106 = 0.94 %). The songs of most crickets were discriminated well across, but also within subfamilies (Figures 6B–F, correctly assigned species were between 90 and 99%). There were only few calling songs within each subfamily that were less well discriminated. This unexpectedly high discrimination was based on the two features used for song discrimination by individual species of crickets (Figure 1): a pulse rate filter based on central timing that differentiates and a duty cycle filter from integration based on a time window (Clemens and Hennig, 2013). LN-models then offer a surprisingly simple view on signal recognition in crickets and the relevant feature space (Figure 5B).

In view of LN-models the bimodal distribution of energy in songs of cricket also suggests two levels of complexity in song pattern recognition (Figure 5B). The recognition of songs with high energy levels composed from long chirps (or trills) will require only a single upper threshold for the computation from the integration time window (Table 1). The discrimination of songs with short chirps would require two such thresholds, a lower one that has to be passed and a higher one that must not be passed for the song to be recognized. The simplicity of the former arrangement is also in line with views on the evolution of song patterns, which suggest that longer chirps (or trills) correspond to the ancestral situation (Alexander, 1962; Otte, 1992; Korsunovskaya, 2008).

In summary, LN-models offer powerful discrimination of cricket songs, based on properties of differentiation (Figure 4B) and integration (Figure 4A). The new feature space indicates bimodal separation of songs by energy or duty cycle, but homogeneous distribution of pulse rates. This view confirms the often used taxonomic criteria of pulse rate, chirp duration (i.e., the number of pulses) and chirp rate as useful discriminators of cricket songs. The recognition of cricket songs can be summarized by a peripheral filter for carrier frequency (Kostarakos et al., 2009), a differentiating pulse rate filter evaluating the temporal song components on a short time scale by the timing of excitation and inhibition (Figure 4) and an integrating filter for song energy (Table 1).

## Consequences for the evolution of communication systems

The goal of the present review was to illustrate the power of a very general coding scheme for sensory processing with only few basic and physiologically plausible components (Table 1). The aim of the following section is to illustrate predictions derived from the general model about filter properties that can be falsified by neurophysiological approaches and to point out consequences for the evolution of communication systems.

### Transitions and transformations between filters

From an evolutionary perspective the properties of Gabor functions suggest a simple solution to the large phenotypic differences observed in the song patterns and preferences of sibling species in different taxa (crickets: *Teleogryllus* Hennig and Weber, 1997, tettigoniids: *Tettigonia*, Schul, 1998, *Neoconocephalus* Schul et al., 2014, *Isophya* Orci, 2007, grasshoppers: von Helversen and von Helversen, 1994). Small changes in strength and timing of excitation and inhibition may already suffice to generate the observed differences (Figures 3, 4). Even the transition from a duty cycle preference to a pulse rate preference may require only few small steps (Figure 4).

### How LN-models may shape the temporal patterns of song signals

A fundamental component of LN-models is the selectivity for temporal characteristics of the pattern that is given by the filter part—in our case the Gabor function (Figures 3, 4, Table 1). The properties of this filter depend on the relative strength and timing of excitation and inhibition that correspond to a fundamental operation performed in sensory pathways in general. The calling songs of crickets, but also other insects, would have to match the filter part of the LN-model over a given integration time window. Consequently a number of different song patterns may suffice to activate the same type of Gabor filter implemented in the auditory pathways of females with very different genetic background. In this view the convergent appearance of song patterns is not surprising (Otte, 1992; Bush and Schul, 2010; Korsunovskaya, 2008). Different mechanisms of signal production may then converge to activate the same type of LN-model as for instance by different layouts of central pattern generators (Marder, 2011) or by the production of the same sound signals due to stridulation with wings or legs within the grasshopper genus *Stenobothrus* (Elsner and Wasser, 1995).

### Simple and complex songs

The songs of many insects, from crickets to katydids and grasshoppers, are of a simple type (crickets: Gryllidae Desutter-Grandcollas and Robillard, 2003, Tettigoniidae: *Neoconocephalus* Bush and Schul, 2010, grasshoppers, Ragge and Reynolds, 1998). The recognition of such song patterns can be described with basic LN-models. However, within all taxa complex songs are also known (crickets: *Teleogryllus* Otte and Cade, 1983), Eneopterinae (Robillard and Desutter-Grandcolas, 2011; Tettigoniidae: Phaneropterinae, Dobler et al., 1994; Walker, 2004; Hemp et al., 2009; grasshoppers: *Chorthippus* Ragge and Reynolds, 1998, *Stenobothrus* Ostrowski et al., 2009). So far it is still unresolved whether more complex patterns in the song signals of insects across very diverse taxa, such as pulse rate sweeps, alternating rhythms or tick and buzz schemes can be explained by the proposed LN-models. The high discrimination of complex songs among the North-American crickets (e.g., *Eneopterinae* in Figure 6A, c.f. Robillard and Desutter-Grandcolas, 2011) by simple LN-models that evaluate only pulse rates and duty cycle is indeed surprising. Several scenarios may account for the evolution of complex songs. In the first case, a complex song may evolve that can be recognized by a simple preference function. For example, the cricket *Teleogryllus oceanicus* exhibits a complex song with two rhythms, but females exhibit a simple preference for a single pulse rate (Pollack and Hoy, 1979; Hennig and Weber, 1997; see also Schul, 1998 for a similar example in katydids). Grasshoppers of the genus *Chorthippus* exhibit elaborate and highly amplitude modulated song signals, but females also respond to simple sound patterns build from blocks of pulses (von Helversen and von Helversen, 1994). In a second scenario, complex songs evolve in response to two or more simple preference functions. For example the cricket *T. commodus* exhibits a song with two pulse rates, both of which have to present for song recognition (Hennig and Weber, 1997), a pattern that is also known for grasshoppers (Stumpner and von Helversen, 1992). Such an atomistic recognition of features build from several simple LN-models may account for alternating rhythms that are known for many species of insects. In a third scenario, the song may be as complex as the recognition similar to a Gestalt-like perception as may be the case for individual neurons in songbirds (Margoliash and Fortune, 1992). Presently, the call recognition of the tettigoniid *Neoconocephalus affinis* is among the most complex known in insects (Bush et al., 2009).

In summary, song signals viewed in the light of sensory processing by a receiver based on LN-models will advance our understanding of how song patterns evolve, how filters shape song signals, how transitions from rate filters to integrating filters are possible and whether simple and complex songs require simple and complex filters. It also allows us to search more specifically for physiological mechanisms. Not at least, LN-models are reminiscent of the technique of Pointillism used in impressionistic paintings. While the sound pulses produced by insects may represent the pixels of different shade and color over time from which all kinds of songs, or acoustic pictures, can be made, the Gabor functions equip us with a pointillistic view on insect songs that touches upon perceptual capacities in much the same way painters did about one hundred years ago.

### Conflict of interest statement

The authors declare that the research was conducted in the absence of any commercial or financial relationships that could be construed as a potential conflict of interest.
